# Impaired renal function and associated risk factors in newly diagnosed HIV-infected adults in Gulu Hospital, Northern Uganda

**DOI:** 10.1186/s12882-015-0035-3

**Published:** 2015-03-31

**Authors:** Pancras Odongo, Ronald Wanyama, James Henry Obol, Paska Apiyo, Pauline Byakika-Kibwika

**Affiliations:** Faculty of Medicine, Gulu University, P.O. Box 166, Gulu, Uganda; Infectious Diseases Clinic, Gulu Regional Referral Hospital, P.O. Box 160, Gulu, Uganda; Department of Medicine, Makerere University College of Health Sciences, P.O Box 7072, Kampala, Uganda

**Keywords:** Renal function, Kidney disease, HIV, Gulu, Northern Uganda

## Abstract

**Background:**

Screening for renal diseases should be performed at the time of diagnosis of human immunodeficiency virus (HIV) infection. Despite the high prevalence of HIV/AIDS in Northern Uganda, little is known about the status of renal function and its correlates in the newly diagnosed HIV-infected individuals in this resource limited region. We aimed to determine the status of renal function and factors associated with impaired renal function in newly diagnosed HIV-infected adults in Northern Uganda.

**Methods:**

This was a seven month cross-sectional hospital-based study, involving newly diagnosed HIV-infected patients, 18 years and older. Patients with history of diabetes mellitus, hypertension and renal disease were excluded. Estimated glomerular filtration rate (eGFR) was calculated using Chronic Kidney Disease Epidemiology Collaboration (CKD-EPI) formula (Table one). Factors associated with impaired renal function (eGFR < 60 ml/min/1.73 m^2^) were thus sought.

**Results:**

We enrolled 361 participants (230, 63.7% female) with Mean ± standard deviation age of 31.4 ± 9.5 years. 52, (14.4%) had impaired renal function (eGFR <60 mL/min/1.73 m^2^) and of this 37 (71.2%) moderate renal impairment (eGFR 30–59.9 mL/min/1.73 m^2^) while 15 (28.8%) had severe renal impairment (eGFR <30 mL/min/1.73 m^2^). Proteinuria was recorded in 189 (52.4%) participants. Of these, 154 (81.5%) had mild (1+) while 8 (4.2%) had severe (3+) proteinuria. Using logistic regression, age, CD4 cell count, and proteinuria were significantly associated with impaired renal function; age >34 years (OR 2.8, 95% CI 1.3 – 5.9; *P* =0.009), CD4 count <350 cells/μL (OR 2.4, 95% CI 1.0-4.7; *P* =0.039) and proteinuria (OR 9.6, 95% CI 5.2–17.9; *P* < 0.001).

**Conclusion:**

The prevalence of impaired renal function was high in new HIV-infected individuals in this region with limited resources. So, screening for renal disease in HIV is recommended at the time of HIV diagnosis.

## Background

Human immunodeficiency Virus (HIV) is a leading cause of morbidity and mortality especially in sub-Saharan Africa. The HIV-infected individuals may suffer both HIV-associated and non HIV-associated kidney disease which may worsen their prognosis. Non HIV-associated kidney disease may follow hypertension, diabetes mellitus, infections and many other causes while HIV-associated kidney disease may occur as HIV-associated nephropathy, nephropathy due to opportunistic infections, immune complex mediated glomerulonephritis, thrombotic microangiopathy, interstitial nephritis and various electrolyte disturbances among others [1-4]. Globally, the prevalence of renal disease among the HIV-infected adult population has been estimated to range between 5 and 30% [5-7]. In sub-Saharan Africa, the prevalence is estimated between 6 and 48.5% [6,8]. In Uganda, the incidence of renal disease in HIV-infected persons is expected to increase, even though highly effective antiretroviral therapy is increasingly available.

Screening for renal disease is recommended for all HIV-infected individuals at diagnosis of the infection and at initiation of antiretroviral therapy (ART) [9]. If not detected and managed timely and appropriately, AIDS-associated renal disease may progress to end-stage renal disease (ESRD) requiring renal replacement therapy [10]. Renal dysfunction at the time of initiation of ART or during ART is associated with complications such as faster progression to AIDS, high blood pressure, bone demineralization and anemia [10] which present significant challenges to HIV management especially in resource limited settings. Early initiation of ART may improve the treatment outcomes of individuals with HIV-associated kidney disease [11].

Although current guidelines recommend determination of renal function prior to ART initiation, this is often not performed due to resource constraints in many areas in sub-Saharan Africa. Moreover, there is enormous evidence that HIV-infected patients receiving ART are surviving longer. Currently recommended ART regimens include 2 nucleoside/nucleotide reverse transcriptase inhibitors (NRTIs) plus a non-nucleoside reverse transcriptase inhibitor as first line therapy and 2 NRTIs plus a protease inhibitor as second line therapy. Some of these drugs are known to cause kidney toxicity such as tenofovir, indinavir and atazanavir, underscoring the need to screen and monitor HIV-infected patients for kidney disease pre and during ART. Several studies have demonstrated ART associated renal toxicity with underlying risk factors such as pre-existing renal disease, untreated and advanced HIV disease, administration of other renal toxic drugs, female gender and older age, longer duration on ART, dehydration, opportunistic infections and African-American ethnicity [12].

Despite the high prevalence of HIV/AIDS in Northern Uganda, little is known about the status of renal function in the HIV-infected individuals in this resource limited region. Renal function pre-ART and during ART is not routinely performed. The cost of chronic renal replacement therapy is exorbitant for majority of people in this setting. An emphasis should be placed on early detection and management of HIV-infected individuals. Interventions such as avoidance of nephrotoxic drugs and substances, blood pressure and glucose control can be used to slow progression and prevent development of end stage renal disease. We aimed to determine the status of renal function and factors associated with impaired function in newly diagnosed HIV-infected adults in Gulu Regional Referral Hospital in Northern Uganda.

## Methods

### Study site

This study was conducted at the infectious diseases clinic of Gulu Regional Referral Hospital between August 2013 and February 2014. The hospital is located in Gulu Municipality in Northern Uganda and supported by the Ministry of Health. All services are provided free of charge to the public including HIV care and treatment.

### Ethical considerations

The study was approved by Gulu University Faculty of Medicine Research and Ethics Review Committee and cleared by Uganda National Council of Science and Technology. All participants provided written informed consent at enrollment. The consent process was administered in the local language.

### Study design and population

We conducted a cross sectional study among newly diagnosed HIV infected adults aged 18 years and above who were not yet receiving cotrimoxazole prophylaxis and/or ART. We excluded patients with conditions known to be associated with kidney abnormalities such as acute infections, pregnancy, previously diagnosed systemic hypertension, diabetes, and known renal disease. Based on these criteria, a total of 23 patients were excluded from this study (4 were hypertensive, 4 had diabetes mellitus type II, 6 had pyrexia (body temperature >38°C), 8 were pregnant and 1 had renal disease). HIV testing was performed using Determine strips (Alere Medical Co, Japan) and confirmed by Stat-pak (Chembio diagnostic system Inc, New York) or Unigold kits. Participants were enrolled consecutively till the required sample size of 365 was achieved.

### Study procedures

Patients who fulfilled the study criteria were enrolled consecutively and interviewed for demographic, social and behavioral data. Medical history was collected and complete physical examination performed. Nutritional status was assessed using body mass index (BMI) which was obtained using the formula body weight in kilograms divided by height squared in meters. Following aseptic technique, 5 mL of venous blood was drawn from each participant into a syringe for renal function tests, and CD4 count. About 1.5 mL of blood for CD4 count was transferred into purple top vacutainers and blood for renal function tests was transferred into orange top vacutainers with a gel separator. Serum creatinine and urea were determined using an automated clinical chemistry analyzer (Humastar 180, Human diagnostics). Estimated glomerular filtration rate (eGFR) was calculated using Chronic Kidney Disease Epidemiology Collaboration (CKD-EPI) formula (Table 1) which studies have shown to be comparable to modification of diet in renal disease (MDRD) for estimating estimated glomerular filtration rate (eGFR) in HIV-infected patients [13].

**Table 1:** **CKD EPI equation for estimating GFR expressed for black race, sex and serum creatinine in mg/dL**

**Sex**	**Serum creatinine, S**_**cr**_ **(mg/dL)**	**Equation (age in years for ≥ 18)**
Female	≤0.7	GFR = 166 × (S_cr_/0.7)^‐ 0.329^ × (0.993)^Age^
Female	>0.7	GFR = 166 × (S_cr_/0.7)^‐ 1.209^ × (0.993)^Age^
Male	≤0.9	GFR = 163 × (S_cr_/0.9)^‐ 0.411^ × (0.993)^Age^
Male	>0.9	GFR = 163 × (S_cr_/0.9)^‐ 1.209^ × (0.993)^Age^

We used clinical guidelines from the US National Kidney Foundation’s Kidney Disease Outcome Quality Initiative (K/DOQI) to categorize renal insufficiency to grade renal impairment [14]. Creatinine clearance of >90 mL/min/1.73 m^2^ was considered normal; creatinine clearance of 60 to 89.9 mL/min/1.73 m^2^ (K/DOQI stage 2) was categorized as mild renal insufficiency; 30 to 59.9 mL/min/1.73 m^2^ as moderate insufficiency (K/DOQI stage 3); and <30 mL/min/1.73 m^2^ as severe insufficiency (K/DOQI stages 4 and 5). For the purposes of this study, eGFR of below 60 mL/min/1.73 m^2^ was used as the cutoff point for impaired renal function [15]. The CD4 cell count was measured using a Facs Calibur CD4 machine. Each participant provided a midstream urine sample for dipstick analysis. The urine samples were analyzed immediately using a dipstick (strips by Alecon cedex, France). Proteinuria on dipstick was reported as negative, 1+ (30 mg/dL), 2+ (100 mg/dL), or 3+ (300 mg/dL) [16].

### Statistical analysis

Data were analyzed using the Statistical Package for the Social Sciences version 16. Social, demographic and baseline clinical and laboratory parameters were summarized into frequencies, Means ± standard deviation (SD). Tests for the significance of association were made using the Pearson chi-square (*χ*^*2*^*)* test for categorical variables and independent sample *t* test for continuous variables. Risk factors for renal impairment were determined with logistic regression. The outcome variable was impaired renal function (eGFR <60 mL/min/1.73 m^2^). The covariates were age, proteinuria, CD4 cell count and serum urea. Risk factors for impaired renal function with *P* values <0.05 during univariate analysis were considered for multivariate analysis using logistic regression to determine independent risk factors of impaired renal function.

## Results

Table 2 summarizes the overall socio-demographic characteristics of the study participants. The same table also shows comparison of these characteristics between the patients with and without renal impairment. Three hundred sixty five (365) ART naïve HIV-infected patients were enrolled into the study. Four patients had incomplete records (age, serum creatinine or gender which we needed to calculate eGFR) and were excluded from the analysis. However, data were missing on occupation for 2 subjects. Of the 361 patients analyzed, 63.7% (230/361) were female. The majority, 90.4% (327/361) of the patients were in early stages of HIV infection (WHO clinical stages I and II). The majority of the patients were non smokers and were not taking alcohol 86.1% (311/361) and 74.9% (274/361) respectively. Very few patients, 5.3% (19/361) had attained education beyond secondary level as the majority had only attained primary education. There were no differences in the socio-demographic categorical variables between the participants with impaired renal function and those with normal renal function as shown in Table 2.

**Table 2:** **General characteristics of the studied population**

**Variable**	**Decreased eGFR <60 mL/min/1.73 m** ^**2**^ **(%)**	**Normal eGFR ≥60 mL/min/1.73 m** ^**2**^ **(%)**	**Total number (%)**	***P*** **value**
**Sex**				0.153*
Female	38 (16.5)	192 (83.5)	230 (63.7)	
Male	14 (10.7)	117 (89.3)	131 (36.3)	
**WHO clinical stage**				0.094*
Early stage (I & II)	44 (13.5)	283 (86.5)	327 (90.6)	
Advanced stage (III & IV)	8 (23.5)	26 (76.5)	34 (9.4)	
**Occupation**				0.442*
House wife/no job	20 (17.1)	97 (82.9)	117 (32.6)	
Peasant farmer	16 (11.3)	126 (88.7)	142 (39.6)	
Employed	15 (15.0)	85 (85.0)	100 (27.8)	
**Residence**				0.453*
Rural	12 (11.4)	93 (88.6)	105 (29.1)	
Urban	40 (15.6)	216 (84.4)	256 (70.9)	
**Smoking**				0.686*
Yes	7 (14.0)	43 (86.0)	50 (13.9)	
No	42 (13.5)	269 (86.5)	311 (86.1)	
**Building type**				0.836*
Permanent	6 (13.6)	38 (86.4)	44 (12.2)	
Temporary	46 (14.5)	271 (85.5)	317 (87.8)	
**Alcohol**				0.872*
Yes	14 (16.1)	73 (83.9)	87 (24.1)	
No	38 (13.9)	236 (86.1)	274 (75.9)	
**Marital status**				0.791*
Married	24 (12.7)	165 (87.3)	179 (49.6)	
Single	7 (13.0)	47 (87.0)	54 (14.9)	
Widowed/Divorced	21 (16.4)	107 (83.6)	128 (35.5)	
**Water source**				0.839*
Hygienic	44 (17.5)	208 (82.5)	252 (69.8)	
Unhygienic	20 (18.3)	89 (81.7)	109 (30.2)	
**Education level**				0.749*
Low (primary & uneducated)	32 (12.5)	219 (87.5)	251 (69.5)	
Medium (secondary)	16 (17.6)	75 (82.4)	91(25.2)	
High (above secondary)	4 (21)	15 (79)	19 (5.3)	
**Household income/month**				0.207*
Low income(<$160)	36 (12.6)	250 (87.4)	286 (79.7)	
Medium income ($160-400)	13 (21.3)	48 (78.7)	61 (17.0)	
High income (>$400)	3 (25)	9 (75)	12 (3.3)	

* ***P*** valve for the Pearson chi-square.

### Renal function, immunological status and nutrition status

A summary of participants’ renal function, immunological status and nutritional status are shown in Table 3. Of the 361 participants, 36.8% (133/361) had impaired renal function. Of these, (81, 60.9%) had mild renal impairment (eGFR 60–89.9 mL/min/1.73 m^2^), 37, 27.8% had moderate impairment (eGFR 30–59.9 mL/min/1.73 m^2^) while 15, 11.3% had severe impairment (eGFR <30 mL/min/1.73 m^2^). Low CD4 cell count (CD4 < 350 cells/μL) was recorded in 38.2% (126/361) of the participants while 33% (119/361) had chronic protein-energy under-nutrition indicated by body mass index (BMI <18.5 kg/m^2^). Proteinuria was recorded in 52.4% (189/361) participants. Of those with proteinuria, majority 81.5% (154/189) had mild (1+) while 4.2% (8/189) had severe (3+) proteinuria.

**Table 3:** **Participants’ renal function, CD4 count, nutritional status and stages of renal damage**

**Variable**	**Number (%)**
**Estimated GFR**	
Normal GFR ≥60 mL/min/1.73 m^2^	309 (85.6)
Reduced GFR <60 mL/min/1.73 m^2^	52 (14.4)
**Stage of CRD** (mL/min/1.73 m^2^)	
No damage >90	228 (63.2)
Stage II 60–89.9	81 (22.4)
Stage III 30–59.9	37 (10.2)
Stage IV 15–29.9	9 (2.5)
Stage V <15	6 (1.7)
**CD4 cell count (cell/μL)**	
High CD4 ≥ 350	227 (62.8)
Low CD4 < 350	126 (38.2)**
**Body mass index (kg/m** ^**2**^ **)**	
<18.5	119 (33)
≥18.5	242 (67)
**Urine protein**	
Negative	172 (47.6)
+ (30 mg/dL)	154 (42.7)
++ (100 mg/dL)	27 (7.5)
+++ (300 mg/dL)	8 (2.2)

**Percentage of participants eligible for ART.

Table 4 shows the Mean ± SD of selected variables and also comparison of means of patients with impaired renal function and those with normal renal function. The Mean ± SD age of the enrolled participants was 31.4 ± 9.5 years. The participants’ Mean ± SD weight and BMI were 55.0 ± 8.8 kg and 19.80 ± 2.8 kg/m^2^ respectively. The median Mean ± SD height CD4 count, eGFR, urea and creatinine were 167.9 ± 7.7 cm, 450.0 ± 130.2 cells/μL, 104.3 ± 45.8 mL/min/1.73 m^2^, 23.5 ± 2.8 mg/dL and 1.2 ± 0.6 mg/dL respectively.

**Table 4:** **Characteristics of study participants at screening by degree of renal impairment**

**Variable, N = 361**	**Mean ± SD of variables**		**Mean ± SD**	***P*** **value**
	**Decreased eGFR <60 mL/min/1.73 m** ^**2**^	**Normal eGFR ≥60 mL/min/1.73 m** ^**2**^		
Age (years)	35.1 ± 8.6	30.7 ± 9.6	31.4 ± 9.5	0.001**
Height (cm)	168.3 ± 7.7	167.6 ± 7.5	167.9 ± 7.7	0.508**
Weight (kg)	56.8 ± 9.3	56.4 ± 8.7	55.0 ± 8.8	0.761**
BMI (kg/m^2^)	19.9 ± 3.1	20.0 ± 2.7	19.80 ± 2.8	0.626**
CD4 count (cells/μL)	334.3 ± 9.7	495.3 ± 130.5	450.0 ± 130.2	<0.001**
eGFR (mL/min/1.73 m^2^)	40.1 ± 15.9	125.2 ± 37.5	104.3 ± 45.8	<0.001**
Urea (mg/dL)	30.1 ± 3.8	22.1 ± 2.5	23.5 ± 2.8	0.039**
Creatinine (mg/dL)	2.76 ± 1.2	0.86 ± 0.2	1.2 ± 0.6	<0.001**

**P valve for the independent sample *t* test.

The mean age of the patients with decreased eGFR was 35.0 years and was significantly higher than 30.67 years for the others, *P =* 0.001. The mean CD4 count (334.27 cells/μL) of the patients with decreased eGFR was significantly lower than that of others 495.28 cells/μL, *P* < 0.001. The mean serum creatinine of patients with decreased eGFR was 2.76 mg/dl and was significantly higher than 0.86 mg/dl for the others, *P <* 0.001. There was no statistical difference in the BMI, weight, height of patients with and without renal impairment (Table 4).

### Factors associated with renal impairment

For logistic regression, moderate renal impairment was used as the outcome variable. The covariates were age, CD4 count, urea, and proteinuria. Age (odds ratio [OR] 2.8, 95% confidence interval [CI] 1.3 – 5.9; *P* =0.009), CD4 count (OR 2.4, 95% CI 1.0-4.7; *P* < 0.039) and proteinuria (OR 9.6, 95% CI 5.2–17.9; *P* < 0.001) were significantly associated with the outcome (Table 5). However, urea was not independently associated with renal impairment (OR 1.9, 95% CI 0.9–4.0; *P* < 0.11).

**Table 5:** **Factors associated with impaired renal function**

**Covariate (N = 361)**	***P*** **value**	**Odds ratio**	**95% Confidence Interval**
Proteinuria	<0.001	9.6	5.2 – 17.9
Age > 34 years	0.009	2.8	1.3 – 5.9
CD4 count <350cells/μL	0.039	2.2	1.0 – 4.7
Serum urea >20 mg/dl	0.11	1.9	0.9 – 4.0

Variable(s) entered on step 1: Protenuria, Age, CD4 count, serum urea.

## Discussion

We aimed to determine renal function status and factors associated with impaired function in newly diagnosed HIV-infected adults in Gulu Regional Referral Hospital in Northern Uganda. Defining renal impairment as eGFR <90 mL/min/1.73 m^2^, we found a high prevalence of renal insufficiency of 36.9%. This finding is similar to that from a study done in Zambia where renal impairment was found in 33.5% of the HIV-infected patients initiating ART [17], and from studies in Nigeria where prevalence was 38% and 39% [8,18] who used the same cut off of eGFR <90 mL/min/1.73 m^2^ to define renal impairment. However, both the Zambian and Nigerian studies used Cockroft and Gault formula for calculating eGFR. When renal impairment was defined as eGFR < 60 mL/min/1.73 m^2^, 14.4% of our newly diagnosed HIV-infected participants had renal dysfunction. Our finding compares very well with the study done in Kenya by Kaloustian *et al.* [19] which recorded a prevalence of 11.5% using the same definition with MDRD formula which compares very well with CKD-EPI. However, our finding is lower than the recent finding of 24% in Nigeria% [18] and higher than findings by Wyatt *et al.* [20] in Rwanda and by Ekat *et al.* [21] in Brazaville, Congo of 2.7% and 7.9% respectively. The low prevalence of renal impairment in Wyatt *et al.* study used a population of only HIV positive women while our study included both men and women. Our finding of 14.4% is almost less than half the finding of Onodugo *et al.* possibly of two reasons. First, most (almost 40%) of the patients with eGFR < 60 mL/min/1.73 m^2^ in the Nigerian study were in chronic kidney disease stage 5 where as only 11.5% of our participants with eGFR < 60 mL/min/1.73 m^2^ were in chronic kidney disease stage 5. It could also be because of the different method used to estimate the GFR in this study. Our present prevalence of 14.4% is almost twice that found in Brazaville yet majority (90.6%) of our participants were in early disease stages (I & II) of HIV infection and majority (70.8%) of the participants in Ekat *et al.* study were in advanced disease stages (III & IV) [21]. The disparity in prevalence between studies seems to be explained mainly by the definitions of the impaired renal function and characteristics of studied patients. There is a chance that the prevalence of renal failure would have been higher than this if we had more of our participants in advanced stages of HIV-infection since the risk of failure increases with advancing clinical stage [22-24].

There was high prevalence of proteinuria among our study participants (n = 189, 52.4%), similar to findings from Nigeria [8]. This finding is higher than that by Chioma *et al.* [25] who found prevalence of proteinuria of 38.1%. The difference could be due to the fact that we used one spot urine sample while Chioma *et al.* used two serial urine samples four weeks apart to diagnose proteinuria. Longo *et al.* [26] found a lower prevalence of proteinuria of 20.5% and Kaloustian *et al.* found much lower prevalence of 6.2% [19]. From our study we observed that proteinuria (3+) detects renal impairment in 100% of the HIV-infected adults who are ART naïve in this resource limited region (Table 3).

Notable in this study is the very high prevalence of mild renal impairment in the study population. Although dose adjustment of medication or additional monitoring may not be necessary in HIV patients with mild renal impairment, and they may improve on antiretroviral therapy, it is important that such patients be identified [14,27]. Mild renal insufficiency has been reported to be associated with increased risk of mortality [28].

For resource limited settings, screening for renal impairment could be limited to individuals who do not immediately qualify for ART. This will detect those that will commence ART due to renal impairment. This because studies have shown that in settings where renal insufficiency is diagnosed but the etiology is unknown; provision of ART in itself improves renal function [29]. This is very important considering the rapid and severe clinical course associated with HIVAN which can improve, with preservation of long-term renal function, by a trial of empiric ART [30].

Studies done in Africa have found renal insufficiency to be associated with increased risk for death [17,19]. We found a significant association between estimated GFR and CD4 cell counts implying that individuals with advanced immune suppression as measured by the CD4 count had more advanced renal impairment as measured by the GFR. Our finding is similar to findings in South Africa, Malawi and India [22-24] where low CD4 cell count below 350cells/μL was an independent predictor for impaired renal function. However, Wyatt et al. did not find a significant association between these two parameters [20]. This high prevalence of renal impairment has significant implications for HIV treatment in this area where tenofovir is the preferred NRTI for first-line ART. Guidelines recommend tenofovir dose adjustments to prevent further renal damage when creatinine clearance is below 50 mL/min [31,32]. Approximately 12% of participants in our study would have required tenofovir dose adjustments and nearly 3% would have benefited from serial creatinine monitoring if they were to initiate tenofovir-based ART. While a strategy of routine screening will increase costs of HIV care in resource constrained settings, it must be balanced against the risk of worsening renal impairment following ART initiation regardless of renal function consideration. Similar concerns should be raised if HIV-infected individuals are to receive other ART drugs with known renal toxicities, such as indinavir and atazanavir.

Majority of our study participants were categorised as WHO clinical stage I and II and had CD4 count greater than 350 cells/μL compared to findings in some studies where ART naïve newly diagnosed HIV-infected patients were found with very low CD4 counts [6,15] signifying advanced immune suppression at start of HIV care. For example in Ekat *et al.* [6], study, 70.8% of HIV-infected adults in advanced disease stage (III & IV) while in our study only 9.4% of the participants in advanced disease stage. Our findings imply that adults in this region possibly seek HIV testing and care services early compared to other settings. Uganda has been implementing the routine counseling and testing program for more than five years. All individuals presenting to a health facility are offered HIV counseling and testing. This could also possibly explain the early HIV diagnosis in this population. More than one third of participants were eligible for ART according to national guidelines at the time the study was conducted which has significant implications for ART provision in Uganda.

Our study had some limitations; both creatinine and proteinuria were measured at a single point in time; therefore, we may have included short term, reversible causes of renal impairment. We did not assess for microalbuminuria although it has been shown to be the earliest marker of renal disease. Furthermore, estimation of eGFR and creatinine clearance is affected by medications, diet, protein intake, circulating cortisol levels, and muscle wasting, factors that this study did not explore.

## Conclusions

The prevalence of impaired renal function was high in HIV-infected individuals in Northern Uganda, a region which is limited resources. So, screening for renal disease in HIV is recommended at the time of HIV diagnosis. We also recommend use of urine dipstick to detect proteinuria which is a good proxy of renal impairment especially moderate (2+) and severe (3+).

## Abbreviations

AIDS: Acquired Immunodeficiency Syndrome
ART: Antiretroviral Therapy
BMI: Body Mass Index
CKD-EPI: Chronic Kidney Disease Epidemiology Collaboration
eGFR: Estimated glomerular filtration rate
GFR: Glomerular filtration rate
HIV: Human immunodeficiency Virus
MDRD: Modified Diet in Renal Disease
NRTIs: Nucleoside/nucleotide reverse transcriptase inhibitors
SD: Standard Deviation
